# Alternative Chemistries for Free Radical-Initiated Targeting and Immobilization

**DOI:** 10.3390/jfb14030153

**Published:** 2023-03-14

**Authors:** Emily T. DiMartini, Christopher J. Lowe, David I. Shreiber

**Affiliations:** Department of Biomedical Engineering, Rutgers, The State University of New Jersey, Piscataway, NJ 08854, USA

**Keywords:** free radicals, stimuli-responsive drug delivery, biopolymers, reactive oxygen species, side effects

## Abstract

Stimuli-responsive biomaterials are an emerging strategy that leverage common pathophysiological triggers to target drug delivery to limit or avoid toxic side effects. Native free radicals, such as reactive oxygen species (ROS), are widely upregulated in many pathological states. We have previously demonstrated that native ROS are capable of crosslinking and immobilizing acrylated polyethylene glycol diacrylate (PEGDA) networks and coupled payloads in tissue mimics, providing evidence for a potential targeting mechanism. To build on these promising results, we evaluated PEG dialkenes and dithiols as alternative polymer chemistries for targeting. The reactivity, toxicity, crosslinking kinetics, and immobilization potential of PEG dialkenes and dithiols were characterized. Both the alkene and thiol chemistries crosslinked in the presence of ROS, generating high molecular weight polymer networks that immobilized fluorescent payloads in tissue mimics. Thiols were especially reactive and even reacted with acrylates in the absence of free radicals, and this motivated us to explore a two-phase targeting approach. Delivering thiolated payloads in a second phase, after the initial polymer net formation, allowed greater control over the payload dosing and timing. Two-phase delivery combined with a library of radical-sensitive chemistries can enhance the versatility and flexibility of this free radical-initiated platform delivery system.

## 1. Introduction

Drug targeting approaches are increasingly investigated to promote drug accumulation within diseased tissues and reduce side and off-target effects [1,2]. Antibody-drug conjugates have been approved by the US Food and Drug Administration (FDA) for certain cancer indications, and targeting is achieved through antibodies that bind upregulated receptors on disease cells [3,4,5,6]. Although this approach has high specificity, the ligand-receptor pairs are unique and the receptors are heterogeneously expressed within diseased tissues, reducing the widespread efficacy of antibody-mediated targeting [7,8]. Stimuli-responsive systems are an intriguing alternative strategy that leverage physiological characteristics to initiate drug accumulation or release. For example, pH, redox status, or enzyme expression are commonly dysregulated in disease [7,9,10]. We have previously demonstrated that polymer crosslinking can be initiated by naturally occurring free radical species to drive the immobilization and accumulation of a coupled payload in situ [11].

Free radicals are highly reactive molecules with an unpaired electron, and reactive oxygen species (ROS) and reactive nitrogen species (RNS) are critical mediators in vivo [12,13]. In healthy tissues, there is a balance between radicals and antioxidants, but in many diseases, the ROS/RNS overwhelm the antioxidants and steal electrons from stable biomolecules [12,14,15]. This oxidative stress contributes to the onset and progression of many diseases, including cancer, neurodegeneration, and autoimmune disorders [12,14,15]. Although high concentrations of radicals are damaging in vivo, free radical reactivity is harnessed in polymer synthesis to initiate crosslinking. Synthetic photoinitiators are widely used to chemically crosslink polymer chains with radical-sensitive terminal chemistries [16]. Polyethylene glycol (PEG) is a popular, bio-inert, FDA-approved polymer backbone, and linear PEG diacrylates (PEGDA) are widely used to form hydrogels [17,18,19,20]. In our previous work, we showed that physiologically-relevant ROS and RNS can crosslink and immobilize PEGDA within collagen tissue mimics [11].

An elevated concentration of free radicals is associated with many diseases that afflict different organs, and tissues are dynamic structures with distinct mechanical and biochemical characteristics [21]. Free radical dysregulation will differ between pathologies and stages of progression. For example, cancer will present with different free radical expression than neurodegenerative disorders, and radical concentrations vary within the disease lifecycle. A versatile system is needed to successfully target a range of tissue types with different radical concentrations. In addition, free radicals exist in healthy tissues at lower levels, but crosslinking would ideally be initiated only in diseased areas. Tissue penetration and crosslinking efficiency can be controlled by changing the initial polymer size and concentration, whereas the reactivity depends on the chemistry [11]. In this paper, we have investigated other terminal polymer chemistries, in addition to acrylates, that react with biological radicals and may confer control over crosslinking in vivo.

Building on our success with ROS/RNS-initiated PEGDA reactions, we explored PEGs with terminal alkenes and thiols, which can undergo homotypic and heterotypic crosslinking upon free radical exposure. Alkene and thiol crosslinking via synthetic radical initiation is well established. Alkenes contain a carbon-carbon double bond, and covalent crosslinks between alkenes are formed by radical addition to the double bond [20]. Thiol-thiol coupling is driven by an oxidation-dependent disulfide formation [22]. Alkenes and thiols cross-react when a radical initiator attacks the thiol group, generating a sulfur-centered thiyl radical that adds to the double bond of the alkene to propagate the radical [23]. Homobifunctional PEG dialkenes and PEG dithiols can be mixed to execute the thiol-ene reaction. Alkenes and thiols occur naturally, which indicates that the polymers will be well tolerated in vivo for stimuli-responsive targeting initiated by physiologically-relevant free radicals.

Introducing alternative terminal polymer chemistries confers flexibility in radical-initiated crosslinking rates, but an added benefit is that these chemistries cross-react in the absence of free radicals. For example, thiols covalently couple with acrylates via a Michael-type addition. This radical-insensitive reaction may enable delivery to be achieved in two distinct phases: (1) radical-initiated polymer net formation and (2) payload delivery via non-radical reactions. We explored a two-stage delivery approach, leveraging unreacted acrylates in a PEGDA network to capture secondarily delivered, thiol-conjugated payloads. Although the primary purpose of this research is to characterize alkene and thiol polymers for targeting and delivery applications, these chemistries are also broadly utilized in biomaterials and tissue engineered systems. These systems inevitably contain unreacted alkenes and thiols, and understanding how these chemistries interact with native radicals can inform the off-target reactions of many bioengineered systems.

## 2. Materials and Methods

Our lab previously established methods to assay the reactivity and immobilization potential of acrylated PEG polymers with ROS [11]. These methods were adapted to characterize the reactivity of alternative terminal chemistries on the polymer backbone. Here, homobifunctional polymers with terminal thiol chemistries are referred to as PEG dithiols, and those with terminal alkene chemistries are referred to as PEG dialkenes. Unless otherwise indicated, all of the experiments were performed in triplicate and the results are reported as the mean ± SD.

### 2.1. Materials

PEG dialkenes (2000 Dalton-HO008008-2K; 5000 Dalton-HO008008-5K; and 10,000 Dalton-HO008008-10K), PEG dithiols (2000 Dalton-HO003003-2K; 5000 Dalton-HO003003-5K; and 10,000 Dalton-HO003003-10K), Rhodamine B-PEG-alkene (FL045008-2K), Rhodamine B-PEG-thiol (FL045003-2K), and 10,000 Dalton PEGDA (HO009009-10K) were purchased from Biopharma PEG Scientific. PEGDA (2000 Dalton-701971) and PEG (2000 Dalton-84,797 and 10,000 Dalton-92897) were purchased from Sigma-Aldrich. Ascorbic acid (255564), 2,2-diphenyl-1-picrylhydrazyl (DPPH-D9132), horseradish peroxidase (HRP-P8375), acetylacetone (P7754), hydrogen peroxide (H_2_O_2_-216763), HEPES (H3537), sodium hydroxide (NaOH-S2770), poly-L-lysine (PLL-P1524), penicillin/streptomycin (P4458), and L-glutamine (G7513) were also purchased from Sigma-Aldrich. The BD TMB Substrate Reagent Set (BDB555214) was purchased from Fisher Scientific. Neurobasal medium (21103-049) and B-27 supplement (17504044) were purchased from Thermo Fisher Scientific. The Vybrant MTT cell proliferation kit (V13154) was purchased from Life Technologies. Amber vials (Verex AR0-9926-13) were purchased from Phenomenex. Type-I bovine collagen (C857) was purchased from Elastin Products Company.

### 2.2. Reactivity with DPPH Radicals

DPPH is a stable free radical commonly used to assay radical scavenging activity. Solutions of PEG dialkene (2000 Dalton), PEG dithiol (2000 Dalton), or thiol-ene (1:1 mix of 2000 Dalton PEG dialkene and 2000 Dalton PEG dithiol) were prepared in picopure water at 6000 μM and diluted serially to achieve the target concentrations. In 96-well plates, 50 μL of 100 μM DPPH in methanol was added to 50 μL of the polymer solutions and incubated for 30 min. The sample absorbance (517 nm) was read on a Tecan Infinite M200 Microplate Reader (Tecan Group Ltd., Männedorf, Switzerland) and normalized to the no-polymer, DPPH-only condition. For positive controls, 2000 Dalton PEGDA [11] and ascorbic acid, a known free radical scavenger, were used.

### 2.3. Reactivity with Oxygen-Derived Free Radicals

H_2_O_2_-derived ROS were used to assess the polymer reactivity with radicals seen physiologically. Equal ratios of H_2_O_2_ and tetramethylbenzidine (TMB) from the BD TMB substrate reagent kit were combined with alkenated, thiolated, and thiol-ene polymers at final concentrations of 0–3000 μM. To catalyze the radical formation, 0.1 μg/mL of HRP enzyme was added, and the reaction was allowed to proceed for 1 min before the addition of a 1 M sulfuric acid stop solution. The absorbance was read at 450 nm and 550 nm, and the 550 nm values were subtracted from the 450 nm measurements to account for any background. Similar to the DPPH assays, the results from polymer-containing wells were normalized to no-polymer controls, and PEGDA and ascorbic acid were included as a benchmark for ROS reactivity.

### 2.4. Crosslinking Characterization with Size Exclusion Chromatography

PEG dialkene and dithiol (2000 Dalton) were reacted with increasing amounts of ROS. Molecular weight post-crosslinking was characterized with gel permeation chromatography (GPC). H_2_O_2_, HRP, and acetylacetone were used to generate stable oxygen radicals, and the concentration of HRP was changed to control the level of radical species present. The samples were prepared at 20 mg/mL polymer in 1X PBS, with 48 mM H_2_O_2_, 120 mM acetylacetone, and either 0, 4, 8, or 12 mg/mL HRP in the final reaction solution. The polymers were reacted with ROS overnight, and the post-reaction samples were diluted 1:10 in GPC column buffer (0.5X PBS with 0.02% NaN_3_) for a final polymer concentration of 2 mg/mL. The diluted samples were pushed through nylon filters into 2 mL amber vials, ensuring a 0.5–1 mL volume of the 2 mg/mL reaction solution in the vial. The samples were loaded into the 1200 Agilent GPC System (UV, RI, and light scattering detectors—miniDAWN TREOS II, Wyatt Technology, Santa Barbara, CA, USA) and run at a 0.5 mL/min flow rate. The Agilent EasiVial PEG/PEO, pre-weighed calibration kit was used to estimate molecular weight. Polymer-only conditions at 2 mg/mL and 1 mg/mL (no HRP, H_2_O_2_, or acetylacetone) and HRP-only conditions were run as controls. The data are reported as the average of three runs.

### 2.5. Polymer Crosslinking in Collagen Gels

Type-I bovine collagen was purchased as a lyophilized powder and reconstituted at 3 mg/mL in 0.02 N acetic acid. Buffered collagen solution was prepared according to the following recipe for 1 mL: 20 μL 50X HEPES, 130 μL 0.15 N NaOH, 100 μL 10X PBS, 73 μL 1X PBS, and 677 μL collagen [24]. HRP was included at 8 mg/mL in the collagen solution. The buffered collagen was kept on ice during preparation, and the solution was plated at 100 μL per well in black, clear-bottom, 96-well plates. The plates were incubated at 37 °C for 2 h to allow collagen to self-assemble into a fibrillar gel. Solutions of PEG dialkene and PEG dithiol were prepared at 10 mM using 2000, 5000, or 10,000 Dalton polymers in 1X PBS. Alkene-PEG-Rhodamine B or thiol-PEG-Rhodamine B was dissolved at 10 mM in picopure water and added at 1% *v*/*v* to the respective polymer solutions. Once the collagen gels had assembled, 100 μL of the polymer solution was added on top of the gel for a final concentration of 5 mM throughout the gel-supernatant volume. As controls, alkene- or thiol-PEG-Rhodamine B was mixed with 2000 and 10,000 Dalton non-functional PEG. The plates were kept at 37 °C overnight for the polymers to penetrate the collagen gels. The following day, H_2_O_2_ and acetylacetone were added to generate ROS (48 mM H_2_O_2_/120 mM acetylacetone in the supernatant volume), and the plates were left at 37 °C overnight to allow ROS-initiated crosslinking to proceed. To remove uncrosslinked polymers from the gels, the supernatant was replaced with 200 μL 1X PBS every 3 h for 5 consecutive washes. The gel fluorescence was measured before (Wash 0) and after (Wash 5) washing with a Tecan Infinite M200 Microplate Reader (Ex: 540 nm/Em: 586 nm), and residual fluorescence was normalized to pre-wash values.

### 2.6. Cytotoxicity and Cellular Protection Assays

To evaluate the cytotoxicity of the alkenated and thiolated polymers and the potential for scavenging-induced cellular protection, the polymers were added to rat cortical neurons with and without radical injury. Cortical neurons were isolated from E18 Sprague Dawley pregnant rats according to the literature techniques under an IACUC-approved protocol previously established in our lab [25]. The neurons were plated at 100,000 cells/well on PLL-coated plates and maintained in Neurobasal media supplemented with 2% B-27, 1% KCl, 1% penicillin/streptomycin, and 0.5% L-glutamine. After 5 days in culture, an H_2_O_2_ screen was performed to determine the radical concentration that reduced the viability by ~40% for the cellular protection studies. The culture media was replaced with B-27-free media containing 0–200 μM H_2_O_2_ and incubated overnight, and the Vybrant MTT cell proliferation assay was performed to measure the neuronal metabolic activity. In separate culture plates, 20 μM H_2_O_2_ was added concurrent with dithiol or dialkene polymers at 0–3000 μM in B-27-free media and incubated overnight. To measure the polymer cytotoxicity, 2000 Dalton PEG dithiol or dialkene was added to cultures at 0–3000 μM (without H_2_O_2_) and incubated overnight. An MTT assay was used to assess the cell viability, and no-cell and no-injury controls were included to account for background and normalize to baseline cell activity.

### 2.7. Two-Stage Immobilization in Collagen Gels

We used the collagen model described above to evaluate a two-stage delivery approach that leverages both the acrylate and thiol chemistries. PEGDA (10,000 Dalton) was delivered to HRP-containing collagen gels, but no fluorescent payloads were added at this stage. H_2_O_2_ and acetylacetone were added to initiate radical formation, and PBS-only and non-acrylated PEG were included as controls. After extensively washing the gels with crosslinked PEGDA, 10 mM Rhodamine B-PEG-thiol was delivered at 0.5, 1, and 2% *v*/*v* in 1X PBS and incubated overnight to allow for reactions with residual acrylates. The gels were washed, and the fluorescence was measured on a Tecan Infinite M200 Microplate Reader.

## 3. Results

### 3.1. Reactivity Assay with DPPH Radicals

**Figure 1:**
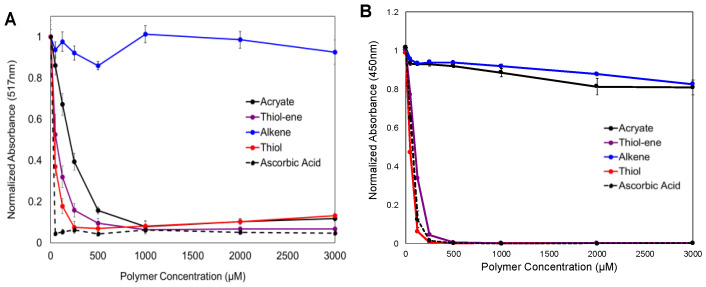
Summary of radical reactivity assays. Reactivity of alkene, thiol, and thiol-ene chemistries with (**A**) DPPH radicals and (**B**) ROS radicals. Acrylates and ascorbic acid were included as positive controls, and a decrease in absorbance indicates reactivity of the different chemistries with the denoted free radical type.

DPPH is a stable free radical that undergoes a colorimetric change upon reduction and is used to measure antioxidant activity spectrophotometrically. Unreacted DPPH radicals appear purple with an absorbance peak at 517 nm. After an antioxidant-induced reduction, DPPH turns yellow, and the absorbance at 517 nm decreases. We characterized the ability of the thiol and alkene PEGs to react with and reduce the DPPH radicals. Ascorbic acid, a known radical scavenger, was used as a positive control of dose-dependent antioxidant activity. The ascorbic acid effectively reduced DPPH, seen as a near complete reduction in absorbance at the lowest antioxidant concentration investigated (Figure 1A). As previously published [11], PEG acrylates reduce DPPH radicals, but a maximum reduction is not reached until a 1000 μM polymer concentration. The PEG dithiols were more efficient reducing agents than the PEG acrylates, although not as efficient as the ascorbic acid control. The PEG dialkenes had limited reactivity with the DPPH radicals, with almost no reduction in the absorbance at high polymer concentrations. The 50:50 PEG dithiol and dialkene mixture efficiently reduced the DPPH radicals, on par with the PEG dithiol-only condition.

### 3.2. Reactivity with Oxygen-Derived Free Radicals

ROS are the most prevalent form of naturally occurring, biological free radical. We measured the polymer reactivity with H_2_O_2_ ROS indirectly using a TMB chromatographic indicator. H_2_O_2_ is catalyzed by HRP to generate free radicals that oxidize TMB. Added polymer chemistries can compete with this reaction to consume radicals. ROS that react with polymer chemistries are unavailable to react with TMB, and this results in a reduction in the TMB color development. Similar to DPPH assays, ascorbic acid was included as a positive control, and the results are shown in Figure 1B. Ascorbic acid efficiently reduced the TMB absorbance, demonstrating that low concentrations of ascorbic acid are able to react with and consume H_2_O_2_ reactive species, and the oxidation of TMB does not proceed. PEGDA performed similarly to the previously published results, with a ~20% reduction in the absorbance at high polymer concentrations [11]. The PEG dithiols had the highest reactivity with ROS of all the chemistries investigated, and the dose-dependent efficiency was on par with the ascorbic acid control. The PEG dialkenes had a similar reactivity to the acrylated PEG polymers, with a dose-dependent reduction in absorbance of up to ~20%. Similar to the DPPH experiments, thiol-ene reactivity resembled the PEG dithiol-only condition.

### 3.3. Crosslinking Characterization with Size Exclusion Chromatography

**Figure 2:**
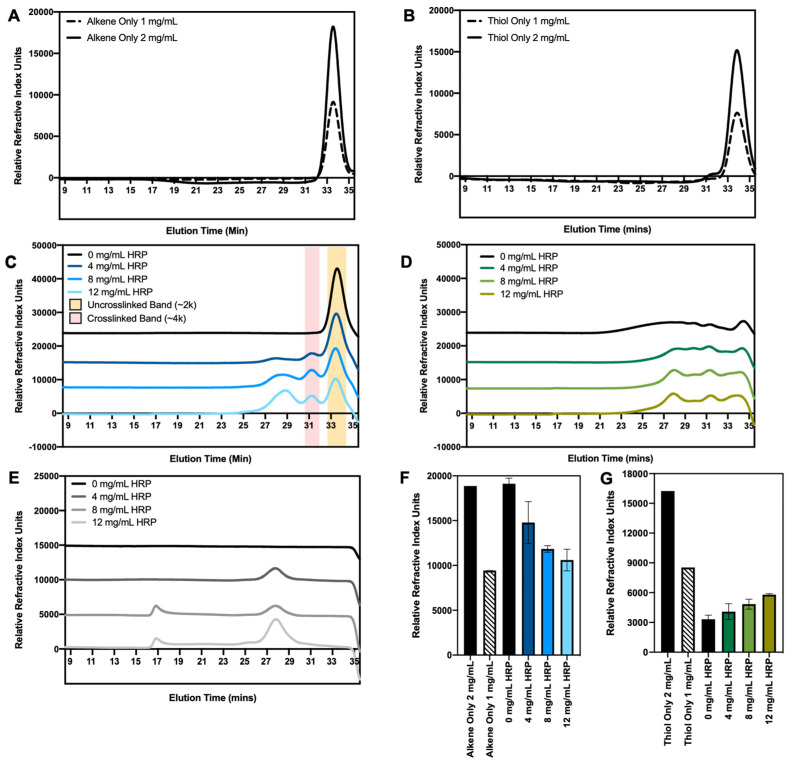
GPC characterization of ROS-triggered size changes in PEG dialkenes and dithiols. (**A**,**B**) 2000 Dalton (**A**) PEG dialkene or (**B**) PEG dithiol were run through the GPC column suspended only in column buffer as controls to establish that peak height corresponds to solute concentration. The 1 mg/mL peak was ~50% of the 2 mg/mL peak, and the 2000 Dalton species eluted the column at ~33.5 min. (**C**,**D**) 2 mg/mL 2000 Dalton PEG (**C**) dialkene or (**D**) dithiol reacted with H_2_O_2_, acetylacetone, and increasing amounts of HRP to control ROS formation. (**E**) Curves representing elution of HRP only at increasing concentrations. (**F**,**G**) Quantification of uncrosslinked peak height for (**F**) alkene curves in (**A**,**C**,**G**) thiol curves in (**B**,**D**).

**Figure 3:**
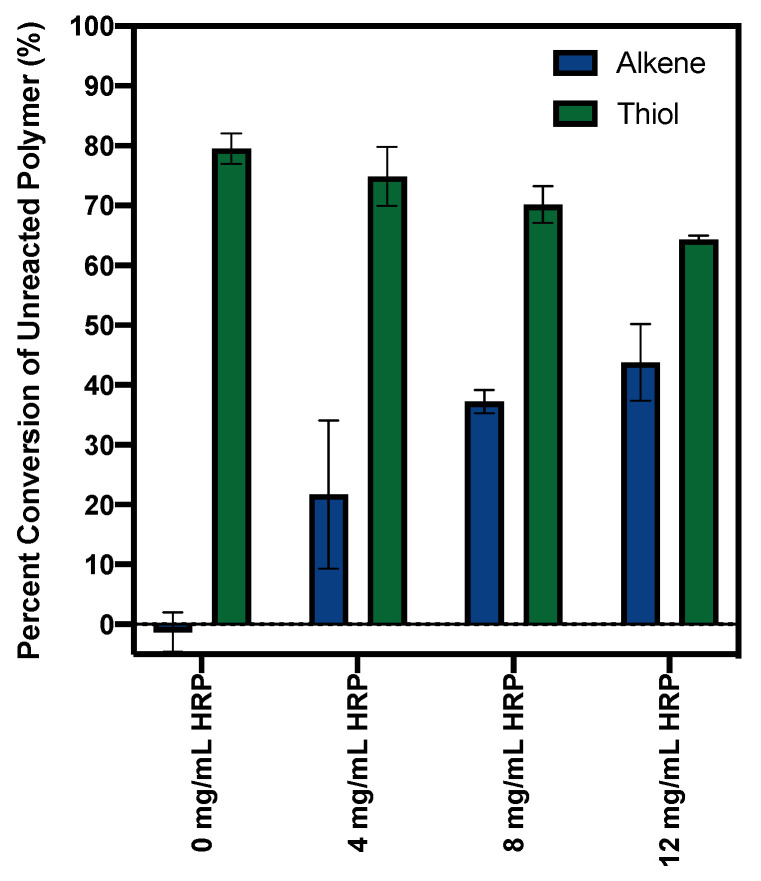
ROS induce alkene and thiol crosslinking, demonstrated as a reduction in the concentration of unreacted PEG. GPC peak heights corresponding to the 2 mg/mL polymer-only controls were quantified for each chemistry to establish the amount of unreacted polymer. In conditions with ROS and PEG dialkene or dithiol, the uncrosslinked peak height was quantified at each HRP concentration. This uncrosslinked peak height was subtracted from and normalized to the 2 mg/mL polymer-only peak to determine the percent of polymers that converted to higher molecular weight species. PEG dialkene followed the expected trend, with negligible conversion when no enzyme was added and a dose-dependent conversion at higher HRP. PEG dithiol had a large conversion even at 0 mg/mL HRP, indicating H_2_O_2_ alone is sufficient in initiating thiol-thiol crosslinks and increasing polymer molecular weight.

In GPC, molecules pass through a column packed with porous beads. Smaller molecules are able to enter the porous material, whereas larger materials are excluded, travel more quickly through the column, and have a shorter elution time. We used this technology to quantify the ROS-initiated crosslinking of the alkenated and thiolated polymers. As a control, 2000 Dalton PEG dialkene alone was run through the column at 1 and 2 mg/mL, and both conditions had a single elution peak at ~33.5 min (Figure 2A). The 1 mg/mL peak was 50.2% of the 2 mg/mL peak, confirming that refractive index (RI) signal intensity correlated to the polymer concentration. In Figure 2C, the dialkenated polymers were reacted with increasing levels of ROS before injection into the column. A single elution peak is seen in the absence of HRP (0 mg/mL), but when HRP is introduced, a new peak emerges at ~31.5 min, indicating that ~4000 Dalton polymer couplets have formed. As the PEG dialkenes convert to higher molecular weights, the height of the unreacted peak decreases, and more reduction was observed as the HRP was increased (Figure 2F). The peaks that emerged ~28.5 min at high HRP concentrations can be attributed to the HRP enzyme (see Figure 2E for HRP-only controls). These experiments were replicated for the dithiolated PEG chemistry, with polymer-only controls in Figure 2B, post-ROS elution curves in Figure 2D, and uncrosslinked peak height in Figure 2G. The polymer-only curves had a single elution peak at ~33.5 min, and the 1 mg/mL peak was 50.3% of the 2 mg/mL height. In contrast to the alkene chemistry, a reduction in the uncrosslinked peak height was observed at the 0 mg/mL HRP concentration (Figure 2G). H_2_O_2_ and acetylacetone alone were able to reduce the uncrosslinked peak height to ~20% of the polymer-only signal. At high HRP concentrations, a new peak emerges at ~31.5 min, corresponding to polymer couplets (Figure 2D). In Figure 3, the percent conversion of the unreacted polymers to the crosslinked species was determined for different HRP concentrations. For the PEG dialkenes, an increase in the HRP concentration resulted in an increase in crosslinking, but the thiolated PEGs had a high level of conversion at all ROS levels, even in conditions without HRP.

### 3.4. Polymer Crosslinking in Collagen Gels

**Figure 4:**
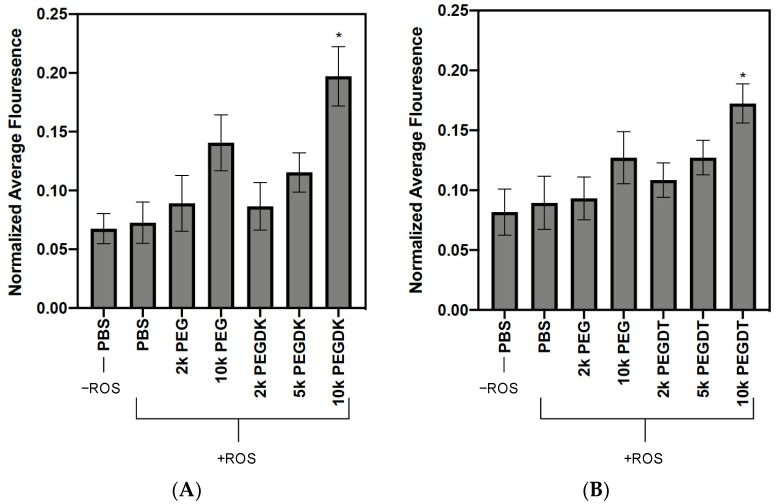
ROS-initiated crosslinking of alkenated and thiolated PEGs immobilizes a payload in collagen gels. (**A**) PEG dialkene and (**B**) PEG dithiol at different molecular weights were added to collagen gels doped 1% with either alkene-PEG-Rhodamine B or thiol-PEG-Rhodamine B, respectively. HRP, acetylacetone, and H_2_O_2_ were subsequently added to generate ROS, and gels were repeatedly washed to remove small, unreacted polymers. Both alkene and thiol PEGs demonstrated a significant (* *p* < 0.001) increase in normalized residual fluorescence for the 10,000 Dalton polymer compared to controls.

To evaluate the potential for crosslinked polymers to remained trapped in tissues, we used collagen gels as a soft tissue mimic. PEG dialkenes and dithiols at various molecular weights were doped with Rhodamine B-labeled alkene or thiol, respectively, where Rhodamine B served as a diagnostic payload to track the delivery. ROS were added to initiate crosslinking, and extensive washing was performed to remove unreacted polymers. A negative control containing only alkene- or thiol-labelled fluorophore was included to establish the baseline diffusion properties, denoted as PBS in Figure 4. PEG polymer controls with no functional groups were included to account for slower fluorophore diffusion due the presence of additional, larger polymers. The residual fluorescence of the PEG dialkenes is shown in Figure 4A and of the thiolated PEGs in Figure 4B. For both chemistries, 10,000 Dalton PEG (non-functional) had elevated residual fluorescence compared to the PBS controls. The PEG dialkenes and dithiols demonstrated a molecular weight-dependent increase in fluorescence, with the 10,000 Dalton condition displaying a significantly higher residual fluorescence than all the other conditions (*p* < 0.001, one-way ANOVA with Tukey post-hoc).

### 3.5. Cytotoxicity and Cellular Protection Assays

**Figure 5:**
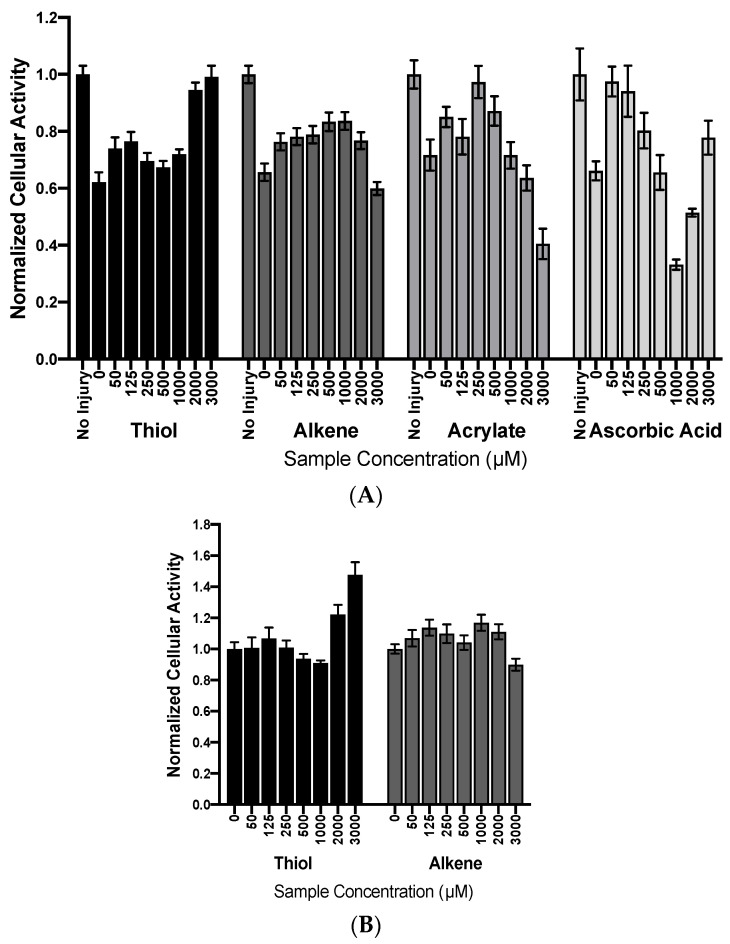
Cellular protection and cytotoxicity. (**A**) H_2_O_2_ induced cell damage in rat cortical neurons, indicted by the decrease in absorbance at 0 μM (no polymer) conditions and measured by a cellular metabolic MTT assay. Thiol and alkene polymers were able to recover cell activity at low polymer concentrations, on par with acrylates. Near complete recovery of cell activity was observed with the ascorbic acid control. (**B**) An MTT assay was used to quantify the dose-dependent toxicity of thiol and alkene polymers on neurons. Both polymers were well tolerated up to 2 mM.

To assess the ability of polymers to react with exogenous radicals and protect cells, rat cortical neurons were cultured and exposed to 20 μM H_2_O_2_, which resulted in a ~40% reduction in the metabolic activity compared to the no-injury condition. Ascorbic acid, a known radical scavenger, fully restored the cell viability at the lowest concentration investigated (Figure 5A). Higher concentrations caused a decrease in the cell metabolic activity, which can be attributed to ascorbic acid toxicity at these levels [11]. Acrylated PEGs were included as a benchmark of polymer-induced protection, and concentrations up to 500 μM resulted in a ~15% increase in cell viability. Similar to ascorbic acid, the decrease in the metabolic activity at high concentrations is related to acrylate cytotoxicity. The PEG dithiols and dialkenes performed similarly to the acrylates, with a ~10% increase in cell metabolic activity at the 50 μM polymer concentration. The alkenes sustained cellular protection up to 1000 μM, with reduced activity at higher concentrations. The thiolated PEGs caused a dip in the cellular metabolic activity around 1000 μM, followed by an apparent increase in the metabolic activity at high concentrations. We also evaluated the cytotoxicity of the thiol and alkene PEGs by introducing polymers directly to the rat cortical neuron cultures (Figure 5B). Neither chemistry substantially impacted the metabolic activity below 500 μM. The PEG dialkenes reduced the cellular activity only at the 3000 μM level, the highest concentration tested. The thiolated PEGs caused a slight decrease in the metabolic activity at 1000 μM, followed by an increase in activity at high concentrations. The changes in metabolic activity in the protection assays (Figure 5A) at high polymer concentrations can be explained by the cytotoxicity assay results.

### 3.6. Two-Stage Immobilization in Collagen Gels

**Figure 6:**
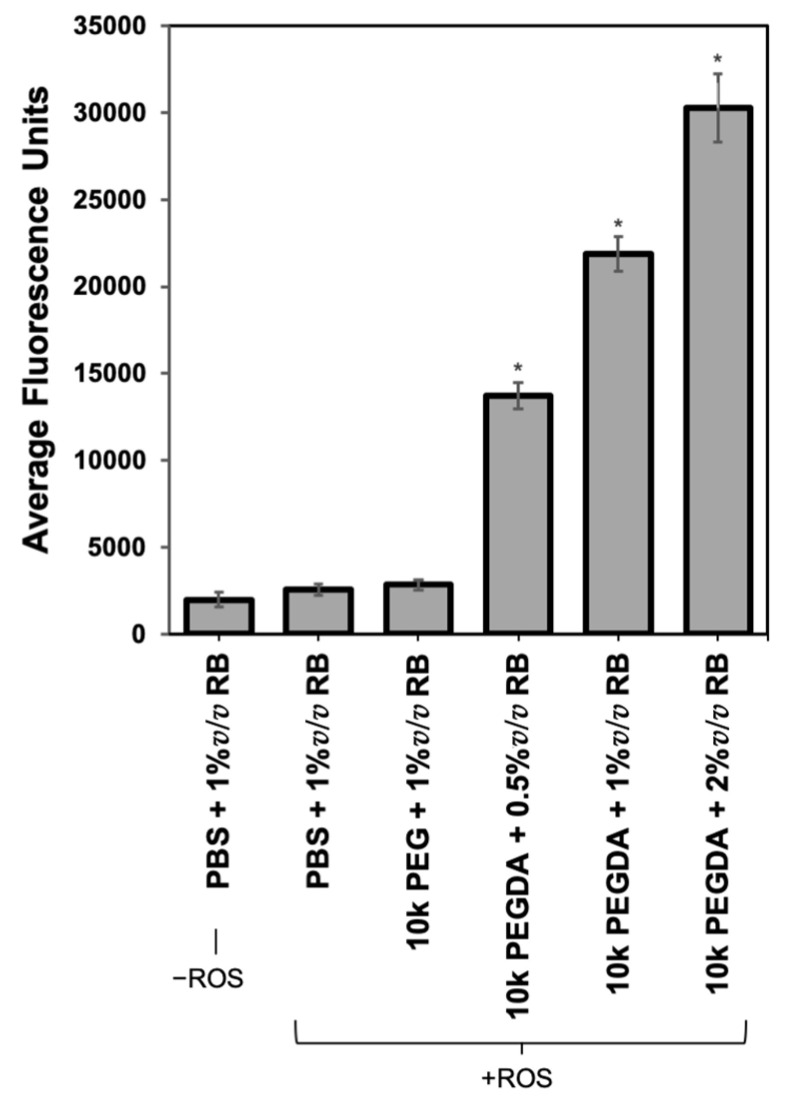
Acrylate and thiol combination system to achieve delivery. PEGDA was crosslinked with ROS in collagen gels, and unreacted polymers were washed out of gels. Subsequently, Rhodamine B (RB)-PEG-thiol was delivered and washed to evaluate two-phase capture. Payload delivery was achieved via the acrylate-thiol reaction, allowing precise dosing control in the second phase (* *p* < 0.001).

Thiols react with acrylates through a radical-insensitive, Michael-type addition, and leveraging the thiol reactivity to administer payloads at later timepoints would allow for more precise delivery. To explore a two-phase approach, PEGDA polymers were crosslinked with ROS in collagen gels, washed, and thiol-PEG-Rhodamine B was delivered in the second stage. Non-functional PEG was delivered in the first phase as a control. Conditions containing PEGDA had a significant (*p* < 0.001, one-way ANOVA with Tukey post-hoc) increase in residual fluorescence after washing (Figure 6), indicating that PEGDA is immobilized within the collagen and that unreacted acrylates are available to capture the thiolated payload. When different fluorophore levels were administered, the payload was trapped at the corresponding doses, demonstrating that the available acrylates were not saturated.

## 4. Discussion

The long-term goal of this research is to develop a stimuli-responsive drug delivery system. Elevated free radicals persist in a wide range of inflammation-associated diseases that occur in different organs, and to successfully target a range of tissue types, each with a unique microenvironment, porosity, architecture, and biochemical profile, a flexible platform targeting system is needed [26]. In a previous study, we demonstrated that ROS can initiate PEGDA crosslinking to target and trap coupled payloads [11]. To build on this success with acrylated polymers and to engineer a more versatile system, we characterized PEG alkenes and PEG thiols as additional building blocks for targeting. Both the PEG dialkenes and dithiols reacted with ROS, resulting in larger, crosslinked polymers that become trapped in collagen gels. The alkenes were comparable to the acrylates, but the thiols, which couple via oxidation-dependent disulfide formation [22], were especially reactive, resulting in non-specific side reactions that are not desirable in practice. However, this high thiol reactivity can also be leveraged through non-radical-initiated reactions with the acrylates, which presented an opportunity to achieve targeting and payload delivery in two distinct phases. The two-phase approach showed great promise to enhance the flexibility and versatility of our drug delivery system. To our knowledge, alkene and thiol crosslinking reactions have not been characterized with ROS initiation for targeting applications.

DPPH and ROS assays were used to assess polymer reactivity with free radicals. The PEG dialkenes were not able to effectively reduce the DPPH radicals, with only a ~10% reduction in absorbance at the highest concentration tested. In the ROS assay, the alkene reactivity was on par with the PEG acrylates, indicating the potential for ROS-initiated crosslinking. The PEG dithiols efficiently reacted with the DPPH and ROS radicals and outperformed the acrylates. This result agrees with previous evidence that thiol-containing cystines are able to consume DPPH radicals on par with ascorbic acid [27]. The thiol-ene condition was also evaluated in the initial free radical assays, but the results indicated that the thiol-thiol reaction predominated, leaving minimal free thiols to react with the alkenes. Based on these screening results, we examined only PEG dithiol and dialkenes (not the thiol-ene chemistry) in the crosslinking and immobilization experiments.

Reactivity assays were used to establish the polymer reactivity, but to successfully target disease sites, radical consumption must result in polymer crosslinking. We used GPC to characterize the molecular weight increases caused by polymer coupling. For the crosslinking and immobilization studies, acetylacetone was included in the ROS generation system to act as a mediator, creating a sustained radical source [28,29,30]. The free radical initiation system and corresponding concentrations were adapted from Gormley et al., and although this range overestimates the radical levels encountered in disease, it serves as a starting point to demonstrate the proof-of-concept for alkene and thiol reactivity with ROS radicals. Polymer-only conditions were run to establish a baseline, and both the PEG dialkenes and PEG dithiols had a concentration-dependent RI signal, with a single, well-defined peak that corresponded to the starting polymer molecular weight (~2000 Dalton). H_2_O_2_, acetylacetone, and 0–12 mg/mL HRP were added to introduce free radicals. For the PEG dialkenes, the 0 mg/mL HRP curve mirrored the alkene-only curve. This is anticipated, as minimal ROS should be generated in the absence of the HRP that catalyzes the reaction. Higher HRP concentrations resulted in the emergence of an RI peak corresponding to a crosslinked polymer product, and a corresponding reduction in the unreacted polymer peak. At 0 mg/mL HRP, for the thiolated PEGs, there was an ~80% reduction in the uncrosslinked signal compared to the polymer-only peak, indicating that H_2_O_2_/acetylacetone alone is able to initiate the conversion to higher molecular weight species. These products formed at a range of higher molecular weights; therefore, a single, detectable peak did not emerge. H_2_O_2_ is a strong oxidizing agent but is not itself a free radical, and previous literature demonstrates that H_2_O_2_ is able to oxidize thiol groups to form disulfide bonds [22,31]. This is corroborated by the high reactivity of the thiols in the initial reactivity screens. The total unreacted polymer conversion did not drastically change at higher HRP concentrations, but a peak corresponding to the PEG dithiol couplets begins to emerge. This indicates that additional ROS in high HRP conditions does drive thiol-thiol polymer crosslinking.

The GPC experiments showed that the radical-initiated crosslinking resulted in higher molecular weight polymers. In the immobilization experiments, we sought to demonstrate proof-of-concept that these polymer networks can become trapped in a matrix-like structure. We used type-I collagen as an in vitro soft tissue model and fluorophore-tagged polymers to track the immobilization. With the addition of ROS, both the 10,000 Dalton PEG dialkene and the dithiol had a significant increase in residual fluorescence compared to the corresponding non-functional PEG. This indicates that the radical-induced crosslinking increased the polymer size and hindered clearance, and polymer immobilization was molecular weight-dependent. The thiolated fluorophores had a higher baseline fluorescence (PBS alone) than the alkenes, but the 10,000 Dalton PEG dialkenes more efficiently sustained the fluorophore compared to the corresponding dithiol condition. There is evidence that highly reactive thiols can interact with type-I collagen molecules, which may account for the increased baseline fluorescence [32]. The GPC studies showed the potential of H_2_O_2_ alone to oxidize thiols and form disulfide bonds, but these bonds are reversible [33]. Thiol side reactions and reversible, H_2_O_2_-dependent oxidation may attenuate bulk payload immobilization in PEG dithiol conditions.

Although the primary goal of this technology is to target a therapeutic or diagnostic payload to a disease site, polymer crosslinking also consumes the damaging free radicals, as evidenced in the DPPH and ROS reactivity assays. Radical consumption has the potential to protect cells from oxidative damage, and this would be a secondary benefit of the delivery system, as there is broad evidence that antioxidant delivery can reduce inflammation and supplement disease treatment [34]. Rat cortical neurons—a primary, sensitive cell type damaged by radicals in central nervous system pathologies—were used for the cellular protection studies. The cells were injured with 20 μM H_2_O_2_, resulting in a ~40% reduction in the cell metabolic activity, as measured by an MTT assay. The thiols, alkenes, and acrylates all provided similar protection, recovering the metabolic activity up to ~15%. We also measured the cytotoxicity of PEG dialkenes and dithiols (no H_2_O_2_) and found that both polymers were well tolerated by the cells at concentrations below 1 mM. Interestingly, high PEG dithiol concentrations increased the absorbance signal. Neufeld et al. conducted cell-free MTT assays with thiol-containing molecules and found that concentrations ranging between 1–100 mM increased the production of the MTT intermediate compared to the controls. The thiols interfered with the MTT signal when no cells were present to reduce the MTT, which suggests that the increased absorbance in cytotoxicity and protection assays at high thiol concentrations was due to thiol interference with the MTT reagent [35]. The initial ROS reactivity assays showed that the thiol reactivity was on par with ascorbic acid, but cellular protection by the PEG dithiol was considerably less than the ascorbic acid control. High thiol reactivity may have resulted in side-reactions with the media and MTT components, and reacted thiols would not be available to consume H_2_O_2_.

We leveraged the high thiol reactivity to demonstrate preliminary evidence for a two-phase targeting system. Thiols participate in Michael-type additions, through which they can couple with acrylates. The reaction mechanism proceeds without free radicals, and this cross-reactivity between the chemistries inspired us to explore a multi-phase targeting approach. We used collagen as our matrix model, paralleling previous experiments. The two-stage immobilization results demonstrated that payload delivery can occur after the initial polymer material is crosslinked. Independently delivering the network-forming polymers and payload-carrying PEGs would allow control over the timing of drug delivery and dose, and multiple, subsequent doses of different drug types can be administered.

## 5. Conclusions

PEG polymers with thiol and alkene terminal chemistries crosslinked in the presence of physiologically-relevant free radicals. This crosslinking consumed the radicals and resulted in polymer nets that prolonged fluorescent payloads in collagen gels. This shows a proof-of-concept for leveraging alkenes and thiols, in addition to acrylates, in a free radical-mediated drug targeting system. Incorporating a library of PEGs with a range of sizes and chemistries can vastly improve the sensitivity and versatility of the system. Thiols had the highest reactivity, with an >80% increase in radical consumption in the ROS assay compared to the alkenes and acrylates, but the associated side reactions may limit the success in free radical-mediated targeting. For example, in the immobilization experiments, 10,000 Dalton PEG dialkenes had a ~4-fold increase in residual fluorescence compared to the baseline, but PEG dithiols only had a 2-fold increase. We leveraged the high thiol reactivity to demonstrate preliminary evidence for a two-phase delivery system through acrylate-thiol coupling. Two-stage targeting employs multiple chemistries to establish distinct crosslinking and delivery phases, which will allow more flexibility in targeting a wide range of disease pathologies.

## 6. Patents

A US patent (US20180360989A1) has resulted from the work reported in this manuscript.

## Data Availability

The data presented in this study are available on request from the corresponding author.
